# Occurrence, Distribution, and Risk Assessment of Phenolic Endocrine-Disrupting Chemicals in Surface Waters of the World’s Longest Water Diversion Project: The Non-Negligible Impact of Local Wastewater Emissions

**DOI:** 10.3390/toxics14050427

**Published:** 2026-05-13

**Authors:** Yuanxin Cao, Xiaoxin Zhang, Yubo Yan, Qiao Li

**Affiliations:** Jiangsu Engineering Research Center of Environmental Functional Materials, School of Chemistry and Chemical Engineering, Huaiyin Normal University, Huai’an 223300, China; y.cao@hytc.edu.cn (Y.C.); yubo.yan@hytc.edu.cn (Y.Y.); qiaoli1218@163.com (Q.L.)

**Keywords:** South-to-North Water Diversion Project, bisphenol A, 4-nonylphenol, 4-tert-butylphenol, water environmental risk

## Abstract

Water contamination by phenolic endocrine-disrupting chemicals (EDCs) is a global environmental concern. Yet, the occurrence of phenolic EDCs in artificial water diversion systems remains poorly understood. Thus, the Eastern Route of the South-to-North Water Diversion Project, the world’s longest water diversion project, was chosen as the study area to investigate the spatiotemporal distribution of alkylphenols (APs) and bisphenol A (BPA), typical phenolic EDCs, and to evaluate their risks. During the diversion operation, higher mean ΣAP concentrations were observed in lakes of Nansi and Dongping relative to the Luoma Lake–Dongping Lake and Yangtze–Luoma Lake diversion channels. The AP composition in the two lakes was also different from that in the two channels. These findings demonstrated that the canal water was not a key source of AP contamination in the lakes, highlighting the importance of local wastewater inputs. The spatial distribution of ΣAP and BPA concentrations in the lakes was mainly affected by the riverine inputs. For ecological risks, 4-n-nonylphenol (4-n-NP) exhibited moderate ecological risks at 81.3% of sampling sites in Dongping Lake and 68.8% of those in Luoma Lake, highlighting the need for heightened attention in future studies. Even under the high exposure scenario, 4-n-NP and BPA would not produce health risks to residents through water intakes. Overall, surface water resources of the Eastern Route Project were confirmed to be safe for human consumption.

## 1. Introduction

To date, approximately 800 chemicals have been identified or hypothesized to disrupt the endocrine system functions in organisms [1,2,3]. Such substances are known as endocrine-disrupting chemicals (EDCs). As a major class of EDCs, phenolic EDCs have attracted extensive research attention in recent decades, as they exhibit estrogenic activity toward wildlife and humans even at trace exposure concentrations [4,5]. Alkylphenols (APs) and bisphenols are typical phenolic EDCs, which are extensively applied in domestic, agricultural, and industrial applications [6,7]. During the stage of production, use, and disposal, APs and bisphenols can be released into the environment [8,9,10,11,12,13]. Therefore, systematic investigation of the occurrence and spatial–temporal distribution of APs and bisphenols in the environment is of great significance for evaluating their potential environmental and health risks.

The APs are phenol derivatives with one or more chains of carbons of varied length attached to the phenol group [14]. Based on the carbon atom numbers in alkyl chains, the APs can be classified into the short (<8 carbon atoms) and long (C8-C12) chain APs. In general, short-chain APs are considered less environmentally problematic than the long-chain APs, due to their higher biodegradability, lower bioaccumulation potential, and lower estrogenic potency [15]. However, 4-tert-butylphenol (4-t-BP), a short-chain AP, warrants increased attention due to its suspected endocrine-disrupting effects [11,16]. Nonylphenols (NPs), a group of substances with a 9-carbon linear or branched alkyl chain attached to phenol, are among the widely used long-chain APs [13,17]. 4-nonylphenol (4-NP), with the alkyl chain located at the para (4-) position on a phenol, exhibits structural similarity to the endogenous estrogens and thus acts as an endocrine-disrupting chemical [13]. Similarly to the 4-t-BP, 4-NP is used in the polymer industry, functioning both as a comonomer for phenolic resins and as a chain terminator in polycarbonate production. In contrast to 4-t-BP, however, 4-NP exhibits different release pathways and exposure patterns [15]. Therefore, the environmental and human risks of the short- or long-chain APs should be assessed individually.

The bisphenols, a category of compounds comprising two phenolic rings, are mainly used to synthesize polymers and resins for subsequent manufacturing of plastic materials [18]. Bisphenol A (BPA) is one of the most extensively used bisphenols, with a worldwide demand exceeding 6.5 million tons in 2012 [19,20,21]. In 2022, China’s in-use stocks were estimated at approximately 34 million tons [22]. Due to the intensive application, as well as inappropriate disposal of BPA-containing products, BPA is continually discharged into the environment [23]. Meanwhile, a large number of studies have confirmed that BPA exhibited endocrine-mediated effects, such as reproductive toxicity, neurotoxicity, and metabolic diseases [10,24]. To safeguard human health, recently, the tolerable daily intake (TDI) of BPA was decreased to 0.2 ng kg^−1^ day^−1^ [25].

APs and BPA are now ubiquitous in aquatic ecosystems worldwide, with their occurrence documented in various rivers and lakes [3,12,26]. The occurrence of APs and BPA in the aquatic environment is closely associated with anthropogenic activities [3,17,27]. Wastewater treatment plant (WWTP) effluents are recognized as a major source of APs and BPA released into aquatic environments [8,28]. The majority of research focusing on the occurrence and distribution of APs and BPA has centered on natural aquatic ecosystems, whereas investigations into APs and BPA residues in artificial aquatic environments remain relatively scarce.

The South-to-North Water Diversion Project is the world’s longest water diversion infrastructure. Comprising three main routes, this project plays a pivotal role in mitigating water scarcity disparities between southern and northern China [29]. The Eastern Route Project was constructed with the purpose of diverting the Yangtze River water to northern China, with a total transfer volume exceeding 1000 million m^3^ during the 2023–2024 operational period [30]. To manage the distribution and diversion of water resources effectively, a series of sluices and pumping facilities were built on the water diversion channels [31]. Previous studies have demonstrated that the distribution of pollutants such as organophosphate esters, microplastics, and neonicotinoids in the channels and lakes of the Eastern Route Project was influenced by the artificial water regulation [32,33,34]. The priority pollutant screening investigation along the Eastern Route Project has revealed the accumulation of toxic organic chemicals in lakes, which may produce potential adverse effects on water-receiving areas [35]. Currently, the occurrence and residue levels of APs and BPA in the Eastern Route Project remain poorly characterized. Notably, water from the Eastern Route Project serves as a potential drinking water source for northern China. Therefore, the assessment of APs and BPA pollution levels in its surface water bodies is critical to safeguarding the safety of the water supply.

Thus, the present study selected the Eastern Route Project as the research area to systematically explore the occurrence and residual levels of APs and BPA in artificial water diversion systems, and to evaluate their associated ecological and human health risks.

## 2. Materials and Methods

### 2.1. Field Sampling

**Figure 1:**
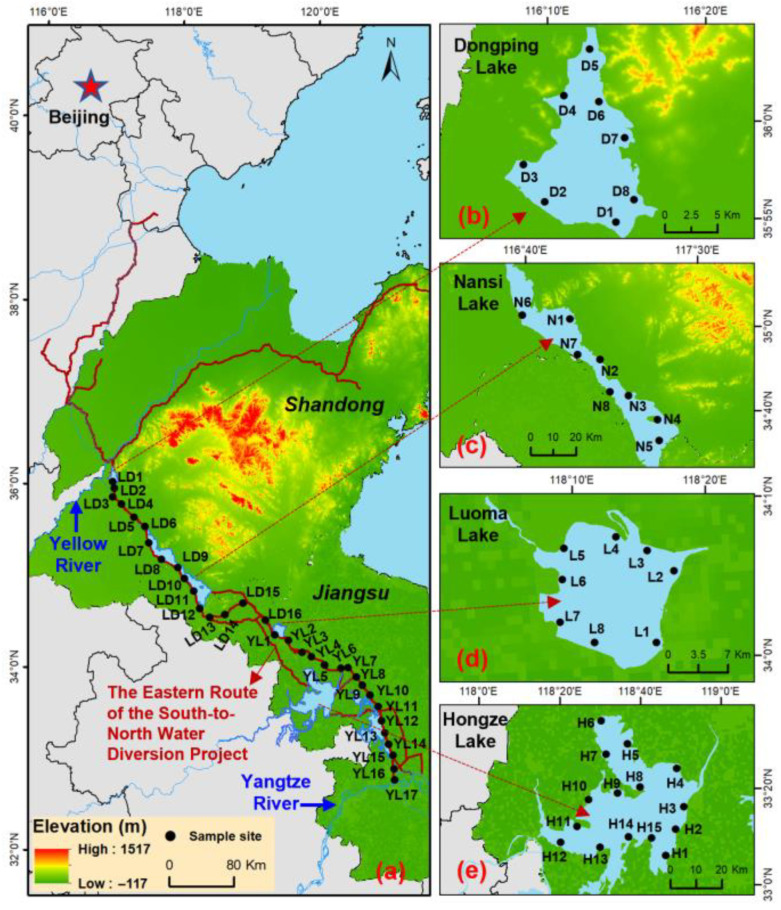
Location of the study area and sampling sites in surface waters of the Eastern Route of the South-to North Water Diversion Project, China. (**a**) Water diversion channels; (**b**) Dongping Lake; (**c**) Nansi Lake; (**d**) Luoma Lake; (**e**) Hongze Lake.

The Yangtze River–Dongping Lake section of the Eastern Route Project, 1045 km in length, is the foundational engineering body for the entire project (Figure 1). This section is divided into two segments: the Yangtze–Luoma Lake (YL) segment and the Luoma Lake–Dongping Lake (LD) segment. Surface water samples (1 L) were collected from 17 sampling sites (YL1–YL17) along the YL water diversion channel in October 2022 and July 2023, and from 16 sites (LD1–LD16) along the LD water diversion channel in July and December 2023. In addition, water samples were taken at 39 sites across the lakes of Dongping (DP), Nansi (NS), Luoma (LM), and Hongze (HZ) in July and December 2023. The precipitation in Xuzhou City, located at the junction of Shandong Province and Jiangsu Province, was reported to be 254.3 mm in July, 8.6 mm in October, and 19.3 mm in December in 2023 [36]. Thus, water samples collected in July were classified as wet-season water samples, while those obtained in October or December were categorized as dry-season ones. Sample collection and storage were performed strictly following China’s National Ecological and Environmental Standards (HJ 1192-2021) [37].

### 2.2. Sample Pretreatment and Analysis

Water sample pretreatment and analysis were conducted in accordance with the aforementioned standard with minor adjustments. Briefly, water samples were filtered through glass fiber filters (0.7 µm, Whatman International Ltd., Maidstone, England) and aliquoted in duplicate. Filtered samples (0.4 L) were subjected to solid-phase extraction at a flow rate of 3 mL min^−1^ using Oasis HLB cartridges (200 mg, 6 mL, Waters Corporation, Milford, MA, USA). The cartridges were eluted with 5 mL of methanol and 5 mL of dichloromethane. The combined eluents were concentrated under a gentle nitrogen stream, filtered through a 0.22 µm PTFE membrane filter, and finally reconstituted in acetonitrile for instrumental analysis. The 9 target APs (4-t-butylphenol (4-t-BP), 4-n-butylphenol (4-n-BP), 4-n-pentylphenol, 4-n-hexylphenol, 4-t-octylphenol, 4-n-heptylphenol, 4-nonyl-branched phenol, 4-n-octylphenol, 4-n-nonylphenol (4-n-NP)) and BPA in the water samples were analyzed by using HPLC-MS/MS. Further details on the instrumental parameters (Table S1), quality assurance, and quality control (Table S2) can be found in the Supplementary Materials.

### 2.3. Risk Assessment

The environmental risks of the target APs and BPA were assessed by the risk quotient (RQ) method [3,34,38], as described below:

(1) $$RQ=\frac{Measured\;environmental\;concentration\;(MEC)}{Predicted\;no\text{-}effect\;concentration\;(PNEC)}$$

The health risks associated with the target APs and BPA were evaluated by calculating chronic daily intake (CDI) (mg kg^−1^ d^−1^) [32,39], as described below:

(2) $$CDI=\frac{APs/BPA\times \;IR\times \;EF\times ED}{\;BW\;\times AT}$$

It was assumed that the drinking water treatment plants removed approximately 80% of the target APs and BPA from surface water [40,41,42,43,44,45]. Thus, the measured concentrations of the target APs and BPA in this study were multiplied by 0.2 for the CDI calculation. *IR* is the water intake rate, which was reported to be 2.0 ± 0.1, 1.7 ± 0.2, 1.4 ± 0.1, and 1.3 ± 0.1 L d^−1^ for men, women, boys, and girls, respectively [46,47]. *EF* is the exposure frequency (365 days yr^−1^). *ED* is the exposure duration, which was suggested to be 9 and 26 years for children and adults, respectively [46,47]. *BW* is the body weight, which was suggested to be 65 ± 3.1, 56.8 ± 3.5, 43 ± 13.3, and 35 ± 11.4 kg for men, women, boys, and girls, respectively [46,47]. *AT* is the average time (365 × *ED*). Considering the variability in body weight and water intake rate, Monte Carlo simulations were introduced in the CDI calculation. The mean values and standard deviations of APs/BPA, IR, and BW, which were assumed to be distributed log-normally, log-normally, and normally, respectively, were utilized to generate 10,000 random data points to calculate the CDI probability distribution. The simulated 90th percentile values were defined as a high-exposure scenario [32].

### 2.4. Statistical Analysis

The statistical analysis of the significant difference was performed by the Tukey–Kramer honest significant difference test at α = 0.05 level, using JMP statistical software packages (JMP 12.0, SAS Institute Inc., Cary, NC, USA) in this study. Monte Carlo simulations were introduced in the CDI estimation by using the Oracle Crystal Ball (version 11.1, Oracle Corporation, Redwood Shores, CA, USA). OriginPro software (version 9.8, OriginLab Corporation, Northampton, MA, USA) was used for the simulation data visualization. Other software such as Microsoft Excel (version 16.0, Microsoft Corporation, Redmond, WA, USA) and SPSS (version 27.0, IBM Corporation, Armonk, NY, USA) were used for data analysis in this study.

## 3. Results and Discussion

### 3.1. Concentrations and Composition of APs in the Eastern Route Project

Nine target APs were analyzed in this study, and only 4-n-BP, 4-n-NP, and 4-t-BP were detected. The detection frequencies of these three APs ranged from 41.2% to 100% in the two water diversion channels, and were 100% in the four lakes (Table S3). This indicated that these three APs were ubiquitous in the lake waters. It has been reported that APs can be transferred into lakes by riverine inputs and atmospheric deposition [14,17,28]. The average ΣAP concentrations in the DP Lake, NS Lake, LM Lake, HZ Lake, LD channel, and YL channel were 233.8, 217.3, 133.1, 117.2, 199.3, and 216.8 ng L^−1^ in the wet season and 155.7, 171.1, 129.2, 109.0, 116.6, and 69.7 ng L^−1^ in the dry season, respectively (Table S3), which were lower than that in the Taihu Lake (486.8 ng L^−1^) [38]. During the diversion operation (dry season), significantly higher mean ΣAP concentrations were observed in lakes of NS and DP relative to the YL channel (*p* < 0.05) (Figure S1).

**Figure 2:**
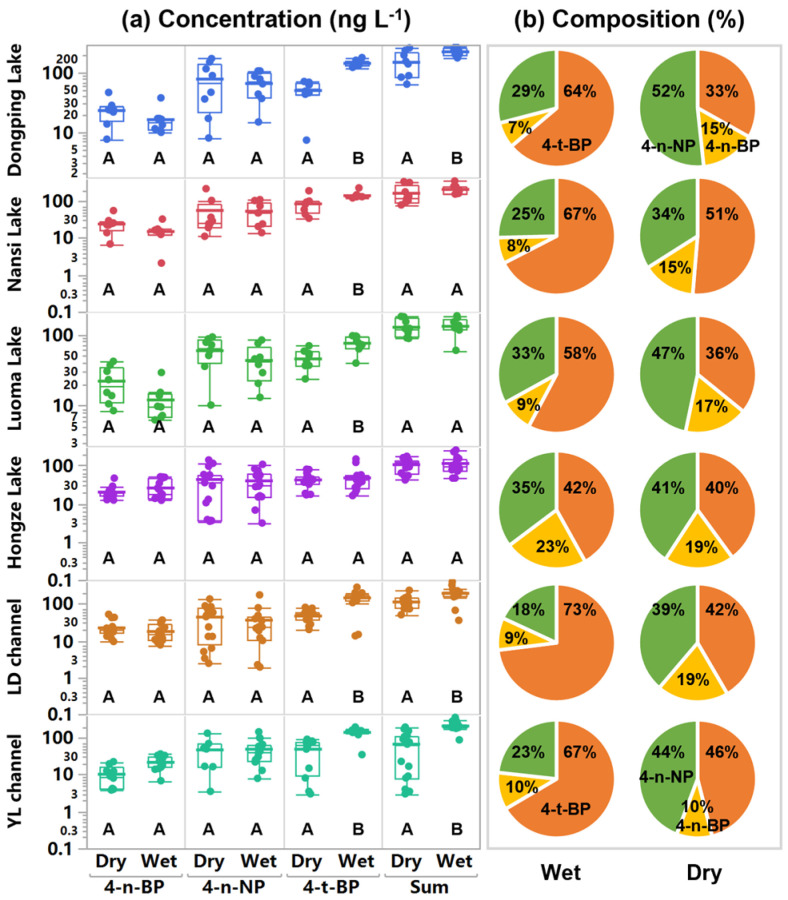
Concentrations(**a**) and composition (**b**) of the targeted alkylphenols (APs) in surface waters of the Eastern Route of the South-to North Water Diversion Project. The difference in AP concentrations between the seasons was significant or not at the 0.05 level for sharing different letters (A, B) or the same letter (A), respectively.

The concentrations in the DP Lake, NS Lake, LM Lake, HZ Lake, LD channel, and YL channel were 7.5–46.6, 2.1–55.3, 6.2–41.8, 12.2–50.6, 7.6–51.5, and 3.8–36.3 ng L^−1^ for 4-n-BP, 7.9–175.2, 11.1–217.6, 10.1–92.2, 3.1–139.2, 1.9–173.3, and 3.5–145.0 ng L^−1^ for 4-n-NP, and 7.2–181.8, 34.2–228.7, 23.6–96.0, 16.2–147.6, 13.4–280.4, 3.0–195.3 ng L^−1^ for 4-t-BP, respectively (Figure 2a; Table S3). The mean 4-n-BP and 4-n-NP concentrations in the channel and lake waters did not exhibit seasonal differences, whereas the average 4-t-BP concentrations demonstrated significant seasonal variations in most locations, excluding HZ Lake (*p* < 0.05) (Figure 2a). The relatively high average concentration of 4-t-BP in the wet season may be related to the high atmospheric deposition and increased surface runoff in the rainy season [11,48]. HZ Lake is the largest regulating reservoir on the Eastern Route Project. The water level changes in the HZ Lake were heavily impacted by human activities, which may have an impact on the distribution of pollutants [32,49].

The average proportions of 4-t-BP in surface waters of the DP Lake, NS Lake, LM Lake, HZ Lake, LD channel, and YL channel were 63.9%, 67.4%, 57.8%, 41.9%, 73.0%, and 66.5% in the wet season, and 33.2%, 51.3%, 35.9%, 40.0%, 41.6%, and 46.1% in the dry season, separately (Figure 2b). The average 4-n-NP proportions in the above locations were 29.1%, 25.4%, 33.0%, 35.3%, 18.1%, and 23.2% in the wet season, and 51.7%, 34.0%, 46.7%, 40.8%, 38.8%, and 44.2% in the dry season, separately (Figure 2b). In DP Lake, the average 4-t-BP proportion decreased by 30.7% from the wet to the dry season, whereas that of 4-n-NP increased by 22.6%, showing the highest seasonal variation among the four lakes studied. As the final level regulating reservoir in the Eastern Route Project, DP Lake received water from Jiangsu Province mainly by the LD and YL channels during the dry season [30]. However, the average concentration and proportion of 4-n-NP in the DP Lake were higher than those in the two channels (Figure 2b). These findings demonstrated that the canal water was not a significant source of 4-n-NP in DP Lake, and the local wastewater emissions cannot be ignored. Numerous prior studies have reported that local WWTP effluents represent the primary source of 4-n-NP in the aquatic environment [11,13,28].

### 3.2. Concentration of BPA in the Eastern Route Project

**Figure 3:**
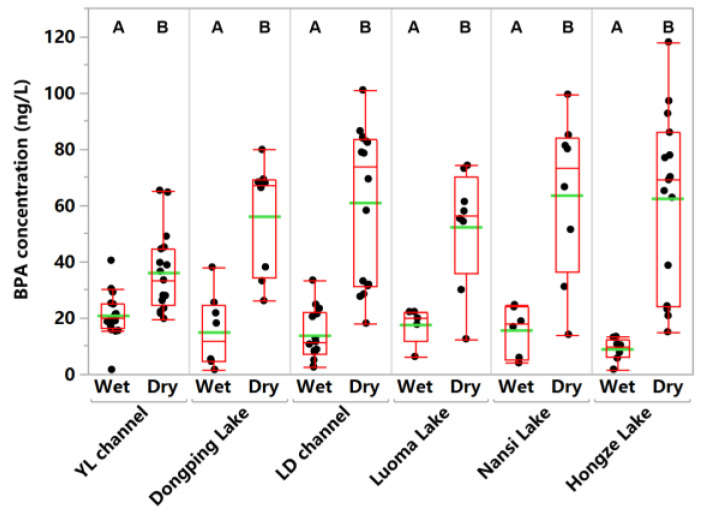
Concentrations of BPA in surface waters of the Eastern Route of the South-to North Water Diversion Project. The difference in BPA concentrations between the seasons was significant or not at the 0.05 level for sharing different letters (A, B) or the same letter (A), respectively.

The BPA detection frequencies in the Eastern Route Project ranged from 53.3% to 100% in the wet season, and were 100% in the dry season (Table S3). This indicated that BPA was ubiquitous in the dry season. BPA may enter the aquatic environment via the production of BPA and BPA-containing products, effluent discharges from WWTPs, landfill leachates, and the leaching of BPA-based materials after disposal [23]. The average BPA concentrations in the DP Lake, NS Lake, LM Lake, HZ Lake, LD channel, and YL channel were 14.9, 15.6, 17.6, 8.9, 13.8, and 20.9 ng L^−1^ in the wet season, and 56.0, 63.5, 52.2, 61.9, 60.9, and 36.0 ng L^−1^ in the dry season, respectively (Table S3; Figure 3). These levels were comparable with those (ng L^−1^) in the Taihu Lake (26.0) [50], Qinhuai River Basin (63.1) [51], and Beiyun River Basin (92.1) [21]. Notably, average BPA concentrations in the aforementioned channels and lakes were lower in the wet season (Figure 3), likely due to the dilution effect of rainfall [48,51]. BPA exhibits negligible volatility and is relatively short-lived in the atmosphere, resulting in a very low concentration in rainfall [10]. At the city scale, a statistically significant positive correlation was identified between mean BPA concentrations in the four lakes and gross domestic product (*p* < 0.05) (Figure S2), implying the impact of socioeconomic activities on BPA contamination in lake waters. With the economic development, the total BPA emissions in the Chinese mainland were reported to be 0.23 Mt in 2022, and approximately 34% of these emissions were released into water bodies [22].

### 3.3. Spatial Distribution of the Detected APs and BPA in the Water

**Figure 4:**
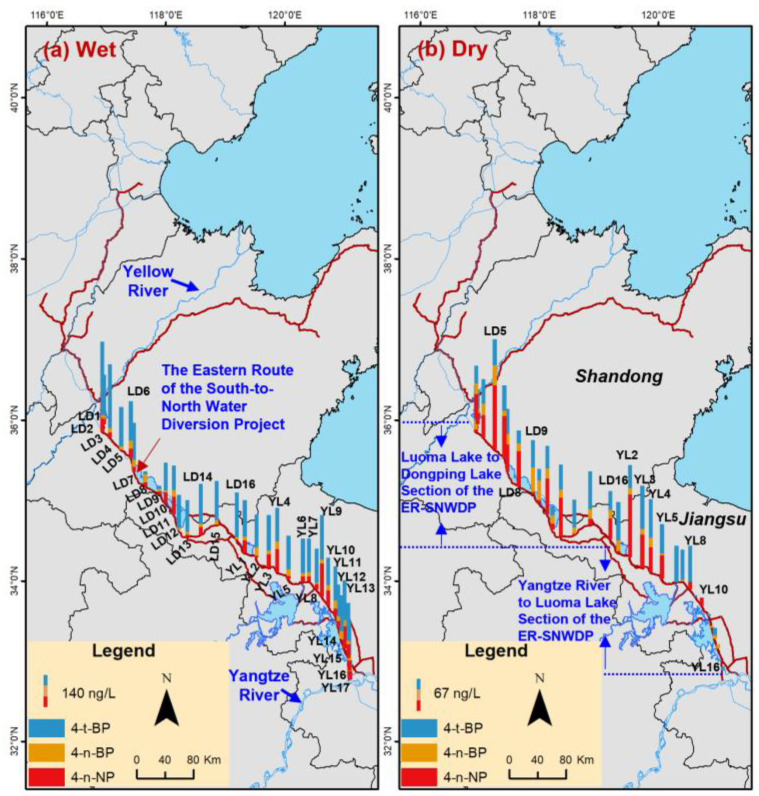
Spatial distribution of the targeted alkylphenol concentrations (ng L^−1^) in the water diversion channels of the Eastern Route of the South-to North Water Diversion Project.

**Figure 5:**
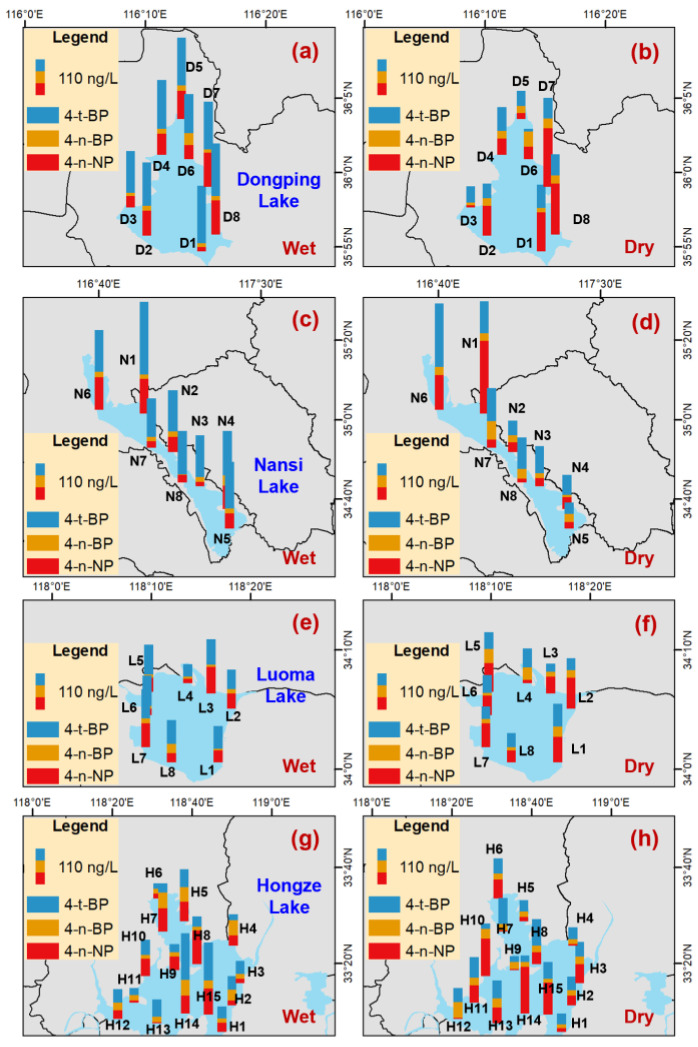
Spatialdistribution of the targeted alkylphenol concentrations (ng L^−1^) in the lakes (Dongping Lake (**a**,**b**), Nansi Lake (**c**,**d**), Luoma Lake (**e**,**f**), and Hongze Lake (**g**,**h**)) of the Eastern Route of the South-to North Water Diversion Project.

In the LD channel during the wet season, the lowest ΣAP concentration was observed at LD9, which was located beside the Weishan sluice (Figure 4a). During the dry season, the AP composition percentage in LD9 was obviously different from that in LD8 (Figure 4b). The sluice operation may hinder the channel water flow, inhibiting the spread of pollution [31]. Thus, the spatial distribution of AP concentrations and compositions in the LD channel may be influenced by the sluice operation. In the YL channel, the lowest ΣAP concentration in the two seasons was observed at YL17, which was located beside the Yangtze River (Figure 4a). During the wet season, the YL channel water would be released into the Yangtze River. The mean annual runoff was reported to be 68.4 m^3^ s^−1^ in the Suqian station of the YL channel [52]. Thus, the YL channel water may be a noteworthy source for APs in the Yangtze River. For lakes during the two seasons, relatively high ΣAP concentrations were observed at D7 and D8 in DP Lake, N1 and N6 in NS Lake, L5 and L6 in LM Lake, and H14 and H15 in HZ Lake, respectively (Figure 5). These sampling sites were located beside the river estuaries. This implied that the spatial variation in AP concentrations within lakes was influenced by the riverine inputs.

**Figure 6:**
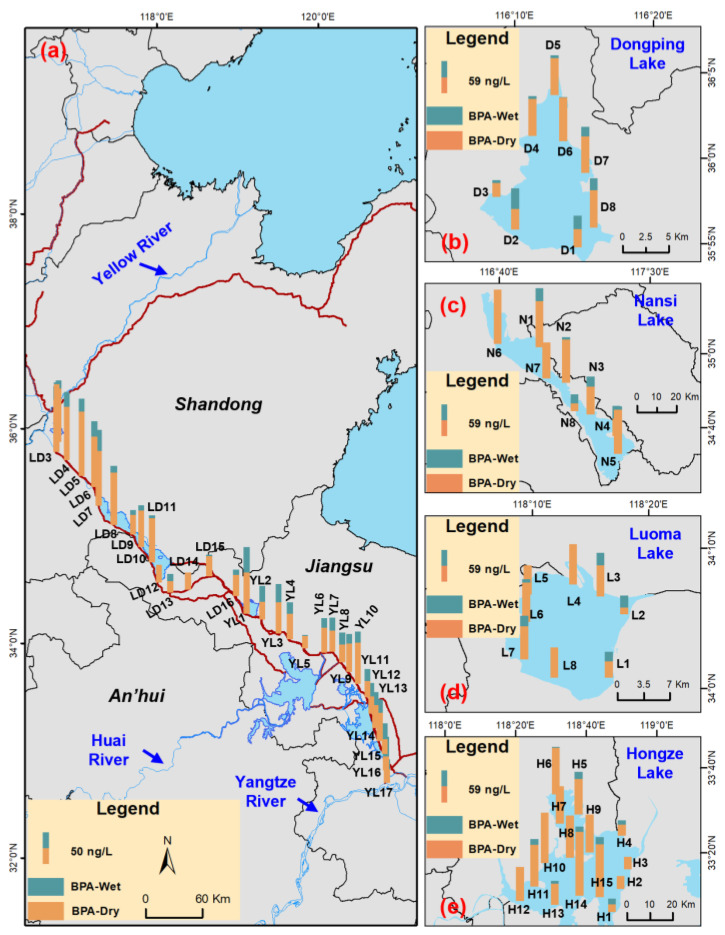
Spatial distribution of BPA concentrations (ng L^−1^) in surface waters of the Eastern Route of the South-to North Water Diversion Project in the dry and wet seasons. (**a**) Water diversion channels; (**b**) Dongping Lake; (**c**) Nansi Lake; (**d**) Luoma Lake; (**e**) Hongze Lake.

In the two seasons, relatively high BPA concentrations in the LD channel were observed at sites of LD3 to LD7 (Figure 6a), which were located beside the Liangshan WWTPs. Thus, the relatively high BPA concentrations in these sites may be related to the WWTP discharges. According to the Shandong Statistical Yearbook in 2023, the total WWTP effluents in Jining City were reported to be 280.6 million m^3^ [53]. The highest BPA concentration in the YL channel in the two seasons was observed at YL1, which may be influenced by discharges from the Pihong River. In the lakes, the lowest BPA concentrations were found at D3 in DP Lake (Figure 6b), N8 in NS Lake (Figure 6c), L2 in LM Lake (Figure 6d), and H2 in HZ Lake (Figure 6e), respectively. These sampling sites, distant from the inflow river estuaries, were subject to limited influence from the fluvial inputs.

### 3.4. Environmental Risk Assessment

**Figure 7:**
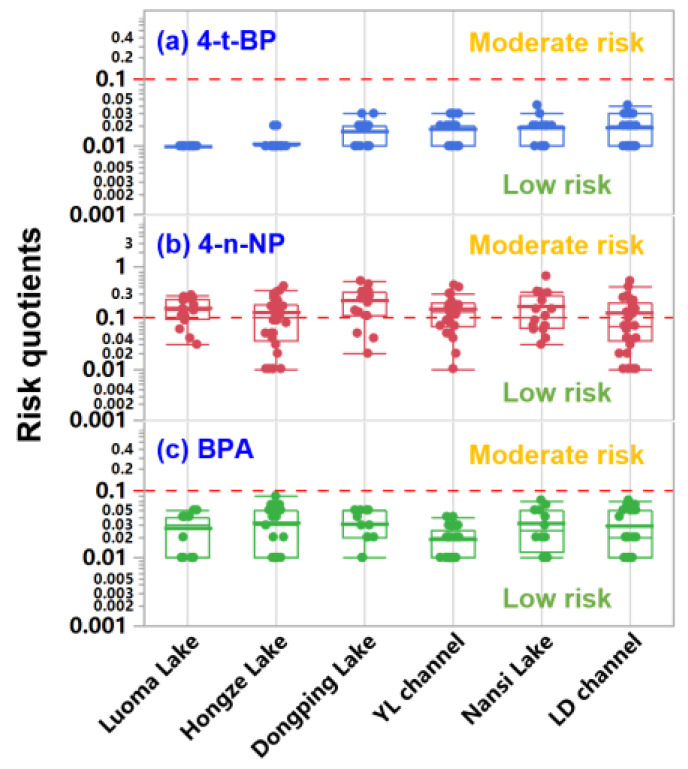
Ecologicalrisks of 4-t-BP (**a**), 4-n-NP (**b**), and BPA (**c**) in surface waters of the Eastern Route of the South-to North Water Diversion Project.

The maximum RQs for 4-t-BP and BPA were below 0.1 in water samples from the two channels and four lakes (Figure 7). This demonstrated that 4-t-BP and BPA posed a low ecological risk [3,51]. The RQs for 4-n-NP were between 0.1 and 1 in 81.3%, 37.5%, 68.8%, 50.0%, 43.8%, and 38.2% of the sampling sites in the DP Lake, NS Lake, LM Lake, HZ Lake, LD channel, and YL channel, respectively, demonstrating that 4-n-NP posed a moderate ecological risk in the studied water bodies. Overall, 4-n-NP in the surface waters of DP Lake and LM Lake needs more attention in future studies. During the risk assessment, the PNEC values for 4-t-BP (6.4 µg L^−1^), 4-n-NP (0.33 µg L^−1^), and BPA (1.5 µg L^−1^) from the European Union were used in this study [15]. These PNEC values were lower than those in China, Japan, and the USA [4,54]. If the PNEC value from China were adopted during the risk assessment, 4-t-BP and BPA would also pose a low risk. At present, the PNEC value for 4-t-BP in China has not been reported. Therefore, the derivation and validation of PNEC values for 4-t-BP in China warrant prioritized research attention.

### 3.5. Health Risk Assessment

**Figure 8:**
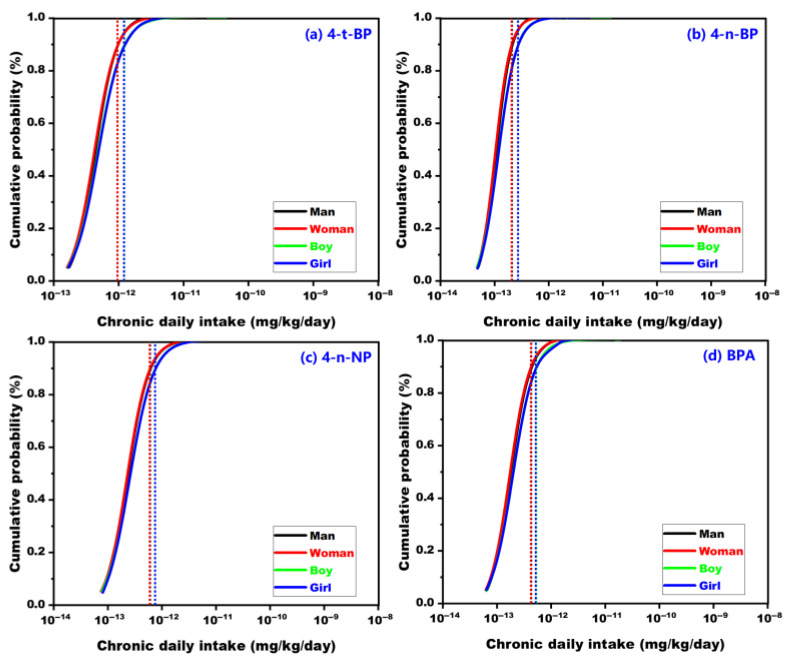
The cumulative probability of estimated chronic daily intake of 4-t-BP (**a**), 4-n-BP (**b**), 4-n-NP (**c**), and BPA (**d**) from water consumption for adults and children. The vertical dashed lines represent the high-exposure scenario CDIs for children and adults.

Surface water from the Eastern Route Project serves as a critical drinking water source for North China. Nevertheless, drinking water treatment plants are unable to achieve complete removal of APs and BPA from such surface water, with removal efficiencies ranging from 60% to 90% [40,41,42,43,44,45]. Therefore, the health risks of the detected APs and BPA by water consumption were evaluated by calculating the CDI. The CDI calculations rely on fixed parameter values. In this study, Monte Carlo simulations were adopted in the CDI calculation by integrating probabilistic distributions of input parameters, enabling a more realistic characterization of exposure risks [32]. The results indicated that the high-exposure scenario CDIs (mg kg^−1^ day^−1^) for the man, woman, boy and girl were 9.6 × 10^−13^, 9.6 × 10^−13^, 1.2 × 10^−12^, and 1.2 × 10^−12^ for 4-t-BP (Figure 8a), 2.1 × 10^−13^, 2.0 × 10−^13^, 2.7 × 10^−13^, and 2.7 × 10^−13^ for 4-n-BP (Figure 8b), 6.1 × 10^−13^, 5.9 × 10^−13^, 7.4 × 10^−13^, and 7.5 × 10^−13^ for 4-n-NP (Figure 8c), and 4.3 × 10^−13^, 4.2 × 10^−13^, 5.3 × 10^−13^, and 5.2 × 10^−13^ for BPA (Figure 8d), respectively. The TDI values for 4-n-NP and BPA in humans were reported to be 5.0 × 10^−3^ and 2.0 × 10^−7^ mg kg^−1^ day^−1^ [15,25]. This suggested that 4-n-NP and BPA in the Eastern Route Project would not produce health risks to residents through water intakes. To date, the TDI values for 4-t-BP and 4-n-BP have not been reported [15], so their health risks cannot be defined. This critical knowledge gap further hinders the formulation of regulatory limits for these two APs in drinking water systems. Therefore, it is necessary for further toxicological studies to determine their TDI values and clarify the corresponding health implications.

## 4. Conclusions

This study focused on investigating the residues of typical APs and BPA in the Eastern Route Project. The findings provide valuable data for understanding the distribution of phenolic EDCs in artificial aquatic environments. The average concentrations of 4-t-BP, 4-n-NP, 4-n-BP, and BPA were 103.0, 45.5, 19.6, and 33.3 ng L^−1^ in the channels, and 75.1, 54.0, 21.0, and 40.5 ng L^−1^ in the lakes, respectively. These levels can serve as a baseline for evaluating the impacts of anthropogenic activities on their levels in the Eastern Route Project. In future studies, more attention should also be focused on the distribution of phenolic EDCs in sediments. The distinct seasonal variations between 4-t-BP and BPA may be attributed to their different input sources. The spatial distribution in concentrations of APs and BPA in the lakes was mainly affected by the riverine inputs. 4-n-NP in DP Lake and LM Lake merits greater focus in subsequent studies. Finally, surface water resources of the Eastern Route Project were confirmed to be safe for human consumption. In future, it is necessary to derive the TDI values for 4-t-BP and 4-n-BP. This will provide a reliable toxicological basis for their health risk assessment.

## Data Availability

The original contributions presented in this study are included in the article/Supplementary Material. Further inquiries can be directed to the corresponding author.

## Supplementary Materials

The following supporting information can be downloaded at: https://www.mdpi.com/article/10.3390/toxics14050427/s1, Figure S1: The total alkylphenol concentration in surface waters of the Eastern Route of the South-to North Water Diversion Project during the dry season. The difference in AP concentrations between the seasons was significant or not at the 0.05 level for sharing different letters or the same letter, respectively; Figure S2: The variations in the average BPA concentrations in four lakes as a function of gross domestic product at city scales; Table S1: Optimized LC-MS/MS parameters for the 9 APs and BPA; Table S2: Recoveries (%) of the 9 APs and BPA in the water, their instrumental limits of detections (LOD) and quantifications (LOQ), and their method limits of detections (MOD) and quantifications (MOQ); Table S3: Concentration ranges (ng L^−1^) and detection frequencies (%) of the target APs and BPA in surface waters of the ER-SNWDP.
