# Supplementary material for: Occurrence, Distribution, and Risk Assessment of Phenolic Endocrine-Disrupting Chemicals in Surface Waters of the World’s Longest Water Diversion Project: The Non-Negligible Impact of Local Wastewater Emissions
Source: Toxics. 2026 May 13;14(5):427. doi: 10.3390/toxics14050427 (PMC13211472; doi:10.3390/toxics14050427)
Supplement: Supplementary file 1 [file toxics-14-00427-s001.zip › toxics-4284176-supplementary.pdf]

Supplementary Material

Occurrence, Distribution, and Risk Assessment of Phenolic Endocrine-Disrupting Chemicals in Surface Waters of the World's Longest Water Diversion Project: The Non-Negligible Impact of Local Wastewater Emissions

Yuanxin Cao, Xiaoxin Zhang *, Yubo Yan and Qiao Li

Jiangsu Engineering Research Center of Environmental Functional Materials, School of Chemistry and Chemical Engineering, Huaiyin Normal University, Huai'an 223300, China; y.cao@hytc.edu.cn (Y.C.); yubo.yan@hytc.edu.cn (Y.Y.); qiaoli1218@163.com (Q.L.)

* Correspondence: xxzhang@hytc.edu.cn

Laboratory analysis

The high-performance liquid chromatography coupled with triple-quadrupole mass spectrometry (HPLC-MS/MS, AB Sciex 4500, USA) was used to analyze the 9 APs and BPA. It was performed in the negative multiple reaction monitoring modes. The injection volume was 10 μL. The mobile phase consisted of two solutions: (A) water with 0.05% ammonium hydroxide, and (B) acetonitrile. Chromatographic separation was running at a flow rate of 0.3 mL min^{-1} under gradient mobile phase condition as follows: 5% B in 2.0 min, 5-20% B in 1.0 min, 20% B in 2.0 min, 20-40% B in 1.0 min, 40% B in 2.0 min, 40-95% B in 1.0 min, 95% B in 3.0 min, returning to 5% B in 0.1 min and kept for 2.9 min. The details of qualitative and quantitative parameters are listed in Table S1.

The analysis is performed under rigorous quality assurance and quality control. The 9 APs and BPA were quantified by using an external standard method. To evaluate the accuracy of the analytical method, recovery experiments were performed by spiking canal and lake water samples at 10 and 50 ng L^{-1}. The recovery of 9 APs and BPA ranged from 70.1% to 93.7% in the channel water samples and from 68.9% to 92.9% in the lake water samples (Table S2). The determined concentrations in this study were not corrected based on the recoveries. Instrumental limits of detection and quantification were set at 3- and 10-fold the signal-to-noise, respectively. Method limits of detection and quantification were in the range of 0.3-0.6 ng L^{-1} and 1.5-2.1 ng L^{-1}, respectively (Table S2). The target compounds were not detected in the solvent blanks, filed blanks, and procedural blanks.

Table S1 Optimized LC-MS/MS parameters for the 9 APs and BPA.

Chemical	CAS No.	Abbreviation	Parent ion m/z, Da	Daughter ion m/z, Da	CE eV	DP eV
4-tert-Butylphenol	98-54-4	4-t-BP	149.2	133.0/117.0	-20/-60	-90
4-n-Butylphenol	1638-22-8	4-n-BP	149.2	106.0/119.0	-15/-35	-90
4-n-Pentylphenol	14938-35-3	4-n-PP	163.2	106.1	-15	-90
4-n-Hexylphenol	2446-69-7	4-n-HP	177.2	106.1/119.0	-18/-40	-90
4-t-Octylphenol	140-66-9	4-t-OP	205.1	133.1/147.0	-25/-40	-90
4-n-Heptylphenol	1987-50-4	4-n-HP	191.2	106.1/119.0	-17/-40	-90
4-nonyl-branched phenol	84852-15-3	4-NP	219.2	106.1	-24	-90
4-n-Octylphenol	1806-26-4	4-n-OP	205.0	106.0	-24	-90
4-n-Nonylphenol	104-40-5	4-n-NP	219.1	133.1/147.0	-35/-30	-90
Bisphenol A	80-05-7	BPA	227.0	212.0/133.0	-24/-31	-90

CE: Collision energy; DP: Declustering potential

Table S2 Recoveries (%) of the 9 APs and BPA in the water, their instrumental limits of detections (LOD) and quantifications (LOQ), and their method limits of detections (MOD) and quantifications (MOQ).

Abbreviation	Channel water		Lake water		LOD	LOQ	MOD	MOQ
	10 ng L^{-1}	50 ng L^{-1}	10 ng L^{-1}	50 ng L^{-1}	ng mL^{-1}	ng mL^{-1}	ng L^{-1}	ng L^{-1}
4-t-BP	77.5 ± 8.2	80.3 ± 4.0	76.3 ± 8.1	77.4 ± 4.5	0.12	0.58	0.39	1.86
4-n-BP	75.5 ± 4.4	73.3 ± 6.7	72.0 ± 2.0	68.9 ± 9.3	0.16	0.57	0.58	2.05
4-n-PP	85.2 ± 1.1	83.3 ± 2.8	75.1 ± 8.3	77.2 ± 8.3	0.18	0.56	0.56	1.76
4-n-HP	89.5 ± 5.3	90.4 ± 1.4	89.3 ± 4.6	87.2 ± 9.4	0.15	0.54	0.43	1.50
4-t-OP	70.1 ± 1.2	71.1 ± 4.8	72.6 ± 3.7	70.5 ± 1.7	0.16	0.53	0.56	1.87
4-n-HP	92.6 ± 5.4	89.4 ± 1.6	92.9 ± 1.3	91.5 ± 3.6	0.11	0.58	0.30	1.59
4-NP	70.9 ± 5.9	74.1 ± 3.6	69.6 ± 0.7	68.9 ± 8.5	0.13	0.53	0.45	1.86
4-n-OP	90.8 ± 2.2	89.3 ± 9.3	86.4 ± 5.1	88.6 ± 2.5	0.14	0.54	0.39	1.52
4-n-NP	80.6 ± 1.3	77.8 ± 6.9	79.3 ± 3.1	76.3 ± 4.9	0.13	0.52	0.42	1.66
BPA	91.3 ± 6.9	93.7 ± 6.0	85.2 ± 7.4	83.1 ± 7.7	0.11	0.53	0.30	1.50

Table S3 Concentration ranges (ng L^{-1}) and detection frequencies (%) of the target APs and BPA in surface waters of the ER-SNWDP.

		4-t-BP	4-n-BP	4-n-NP	ΣAP	BPA
Dongping Lake 2023.8 Wet	Mean	149.3	16.6	67.9	233.8	14.9
	Min	124.0	9.8	14.7	179.8	1.5
	Max	181.8	37.7	108.0	285.1	37.9
	SD	18.4	9.0	34.1	35.4	13.0
	CV (%)	12.3	54.3	50.2	15.1	86.8
	DF (%)	100	100	100		100
Dongping Lake 2023.12 Dry	Mean	51.6	23.6	80.4	155.7	56.0
	Min	7.2	7.5	7.9	64.4	26.0
	Max	71.5	46.6	175.2	265.9	79.7
	SD	20.9	11.4	63.4	75.0	20.3

	CV (%)	40.5	48.4	78.8	48.1	36.2
	DF (%)	100	100	100		100
Nansi Lake 2023.8 Wet	Mean	146.5	15.6	55.2	217.3	15.6
	Min	122.2	2.1	13.9	155.2	3.9
	Max	228.7	32.6	109.6	352.3	24.6
	SD	34.1	8.4	37.1	66.4	8.8
	CV (%)	23.3	53.7	67.2	30.6	56.6
	DF (%)	100	100	100		75
Nansi Lake 2023.12 Dry	Mean	87.8	25.1	58.2	171.1	63.5
	Min	34.2	6.9	11.1	78.8	14.0
	Max	191.2	55.3	217.6	338.2	99.3
	SD	48.8	14.2	70.7	102.2	29.2
	CV (%)	55.5	56.4	121.5	59.7	46.0
	DF (%)	100	100	100		100
Luoma Lake 2023.8 Wet	Mean	76.9	12.2	44.0	133.1	17.6
	Min	39.5	6.2	13.0	59.7	6.2
	Max	96.0	29.1	83.5	183.4	22.1
	SD	19.3	7.6	24.8	38.1	6.6
	CV (%)	25.1	61.8	56.5	28.6	37.7
	DF (%)	100	100	100		62.5
Luoma Lake 2023.12 Dry	Mean	46.3	22.5	60.3	129.2	52.2
	Min	23.6	8.4	10.1	87.9	12.5
	Max	69.3	41.8	92.2	179.3	74.1
	SD	14.5	12.6	27.7	36.5	21.1
	CV (%)	31.3	56.1	45.9	28.2	40.4
	DF (%)	100	100	100		100
Hongze Lake 2023.8 Wet	Mean	49.2	26.7	41.3	117.2	8.9
	Min	16.2	12.2	3.1	46.6	1.6
	Max	147.6	50.6	106.8	253.1	13.3
	SD	36.8	15.1	30.4	61.6	3.9
	CV (%)	74.9	56.6	73.5	52.6	43.7
	DF (%)	100	100	100		53.3
Hongze Lake 2023.12 Dry	Mean	43.5	20.9	44.5	109.0	61.9
	Min	17.0	12.4	3.5	41.7	15.0
	Max	79.7	46.5	139.2	173.6	110.9
	SD	19.6	8.5	43.1	42.1	30.6
	CV (%)	45.0	40.5	96.8	38.6	49.4
	DF (%)	100	100	100		100
YL Channel 2023.8 Wet	Mean	146.2	22.5	51.1	216.8	20.9
	Min	34.8	6.8	7.9	87.6	1.6
	Max	195.3	36.3	145.0	357.4	40.3
	SD	32.7	8.6	36.9	58.2	8.1
	CV (%)	22.4	38.1	72.3	26.9	39.0
	DF (%)	100	100	94.1		100
YL Channel 2022.10 Dry	Mean	50.7	10.7	48.7	69.7	36.0
	Min	3.0	3.8	3.5	3.0	19.7
	Max	91.0	22.4	132.7	194.7	65.2
	SD	33.9	6.8	44.0	67.0	14.0
	CV (%)	66.8	63.4	90.3	96.1	38.9
	DF (%)	70.6	52.9	41.2		100.0
LD Channel	Mean	150.7	18.3	37.3	199.3	13.8

2023.8 Wet	Min	13.4	7.6	1.9	35.3	2.5
	Max	280.4	36.0	173.3	395.6	33.3
	SD	65.7	9.7	45.7	84.4	9.3
	CV (%)	43.6	53.0	122.6	42.4	67.6
	DF (%)	100	100	81.3		87.5
LD Channel 2023.12 Dry	Mean	48.5	22.9	45.2	116.6	60.9
	Min	19.9	9.4	2.4	50.1	18.0
	Max	79.8	51.5	134.7	231.8	100.8
	SD	16.6	11.9	38.7	42.9	27.6
	CV (%)	34.2	52.2	85.7	36.8	45.3
	DF (%)	100	100	100		100

SD: standard deviation; CV: coefficient of variation; DF: detection frequencies; LOD: the limits of detection.

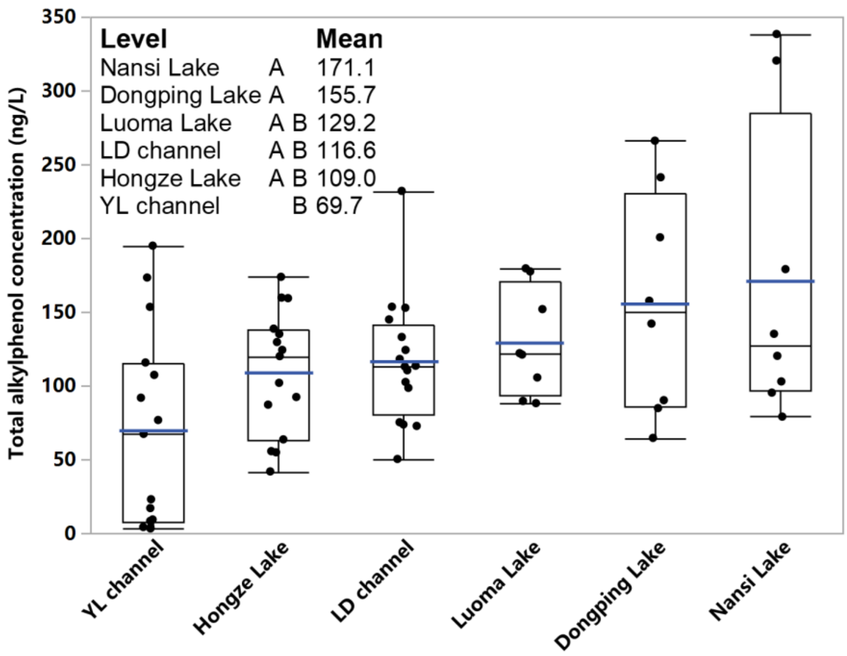

Figure S1. The total alkylphenol concentration in surface waters of the Eastern Route of the South-to North Water Diversion Project during the dry season. The difference in AP concentrations between the seasons was significant or not at the 0.05 level for sharing different letters or the same letter, respectively.

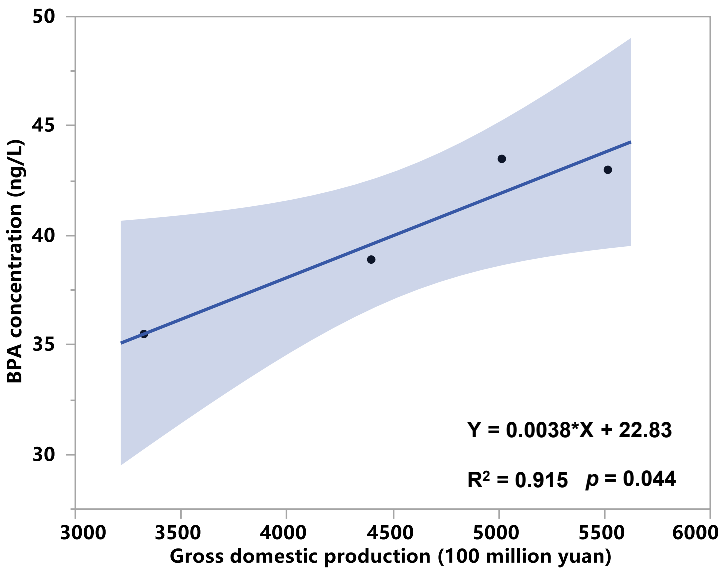

Figure S2. The variations in the average BPA concentrations in four lakes as a function of gross domestic product at city scales.
